# *In Vivo* Analysis of Trapeziometacarpal Joint Kinematics during Pinch Tasks

**DOI:** 10.1155/2014/157295

**Published:** 2014-02-10

**Authors:** Li-Chieh Kuo, Chien-Ju Lin, Guan-Po Chen, I-Ming Jou, Chien-Kuo Wang, Irina G. Goryacheva, Marat Z. Dosaev, Fong-Chin Su

**Affiliations:** ^1^Department of Occupational Therapy, National Cheng Kung University, Tainan 701, Taiwan; ^2^Department of Biomedical Engineering, National Cheng Kung University, Tainan 701, Taiwan; ^3^Musculoskeletal Research Center, National Cheng Kung University, Tainan 701, Taiwan; ^4^Department of Orthopedics, National Cheng Kung University, Tainan 701, Taiwan; ^5^Department of Radiology, National Cheng Kung University Hospital, Tainan 704, Taiwan; ^6^Institute for Problems in Mechanics, Russian Academy of Sciences, Moscow 119526, Russia; ^7^Institute of Mechanics, Lomonosov Moscow State University, Moscow 119992, Russia; ^8^Medical Device Innovation Center, National Cheng Kung University, Tainan 701, Taiwan

## Abstract

This study investigated how the posture of the thumb while performing common pinch movements and the levels of pinch force applied by the thumb affect the arthrokinematics of the trapeziometacarpal joint *in vivo*. Fifteen subjects performed the pinch tasks at the distal phalange (DP), proximal interphalangeal (PIP) joint, and metacarpophalangeal (MP) joint of the index finger with 0%, 50%, and 80% of maximal pinch forces by a single-axis load cell. 3D images of the thumb were obtained using the computed tomography. The results show that the reference points moved from the central region to the dorsal-radial region when changing from pinching the DP to the MP joint without pinching force being applied. Pinching with 80% of the maximum pinching force resulted in reference points being the closest to the volar-ulnar direction. Significant differences were seen between 0% and 50% of maximum pinch force, as well as between 0% and 80%, when pinching the MP joint in the distal-proximal direction. The effects of posture of the thumb and applied pinch force on the arthrokinematics of the joint were investigated with a 3D model of the trapeziometacarpal joint. Pinching with more than 50% of maximum pinch force might subject this joint to extreme displacement.

## 1. Introduction

Human hands play an important role in performing various daily activities, most of which involve grasping and pinching actions. Adequate joint flexibility, sufficient muscular strength, and appropriate hand sensations are required to accomplish coordinated thumb-finger interactions [1, 2], and a well-integrated thumb-finger relationship is regarded as a crucial element in a variety of tasks which are based on power grip or precision pinch performance [3–5]. Among the numerous factors that contribute to such performance, a proper thumb, with either structural or functional integrity, is one indispensable component. More specifically, the multidirectional and wide motion ranges of the thumb are essential elements in activities requiring grasp and pinch functions. The trapeziometacarpal (TMC) joint, located at the base of the thumb, is one with saddle forms which allows great mobility and thus is of particular importance [6, 7].

In the last two decades, many researchers have investigated the biomechanical characteristics of the TMC joint and tried to develop some objective assessments for quantifying its kinematics [8–17]. The range of motion, center of rotation (COR), working space, joint stability, and joint contact pattern of the TMC joint have been reported in these studies. The results indicate that the average range of motion of the TMC joint is from 41° to 47° in flexion-extension, from 53° to 63° in abduction-adduction, and approximately from 40° to 50° in axial rotation [4, 9, 10]. As a consequence of its unique anatomical configuration, some specific and compound movements, such as opposition and circumduction, can be produced by motions from different planes of this joint, even if the range of joint motion in each plane is not especially large. Due to the great mobility that the TMC joint possess, its stability is an important issue [13, 18]. During thumb movements, the stability of this biconcave saddle joint is mainly provided by the ligaments around the joint, and an injured or unstable TMC joint would have an adverse impact on hand functions when performing most of activities.

Due to the possibility of capsular laxity, a balance between mobility and stability at the TMC joint is required to perform movements effectively [19]. In the studies regarding joint stability, the joint gliding distance is often used to describe joint laxity and congruency. Previous research designed a tester to examine the joint laxity of the TMC joint during lateral pinch, and the results showed that ligament reconstruction and osteotomy significantly affected this [18]. In order to explore joint laxity *in vivo*, X-ray images have been used to estimate the distance of radial gliding of the TMC joint among normal subjects, as well as patients with osteoarthritis (OA), while taking a thumb stress-view test [17]. Clinically, OA in the TMC joint commonly results from its great range of motion and either high or repetitive compressive force [19, 20]. OA in the TMC joint with OA may adversely affect pinch and grasp performance due to the increased pain, reduction in strength, and decrease in the range of motion. It has been reported that the average compressive force at the TMC joint is 12 kg when 1 kg of pinch force is applied during a pinch performance [7]. Although pinching is a common and important movement, the impacts of pinch locations, as well as the amount of pinch forces on the TMC joint kinematics *in vivo*, have seldom been discussed. A better understanding of the characteristics of this joint when performing pinching tasks may have practical benefits with regard to providing improved joint protection strategies while using hand tools and determining the pathomechanism underlying OA. Consequently, the purpose of this study is to explore the TMC joint kinematics while performing lateral pinch tasks when using different thumb postures and applied forces, using a 3D model *in vivo*. This study hypothesized that different postures of the thumb as well as the applied pinch force while performing the pinching tasks might have the effects on the kinematic characteristics of the TMC joint.

## 2. Materials and Methods

### 2.1. Subjects

The subjects in this study were fifteen healthy, right-handed male volunteers, with ages ranging from 20 to 40 years old (23.9 ± 3.9 years) and body heights and weights of 175.93 ± 5.64 cm and 70.13 ± 8.56 Kg, respectively. None of them had previous injuries or prior concerns with regard to the hand and especially the joints of the thumb. All the subjects were informed about the purpose of this study and signed consent forms, and this work was approved by the Institutional Review Board (no: ER-99-112) of the National Cheng Kung University Hospital, Taiwan.

### 2.2. Acquisition Systems

**Figure 1:**
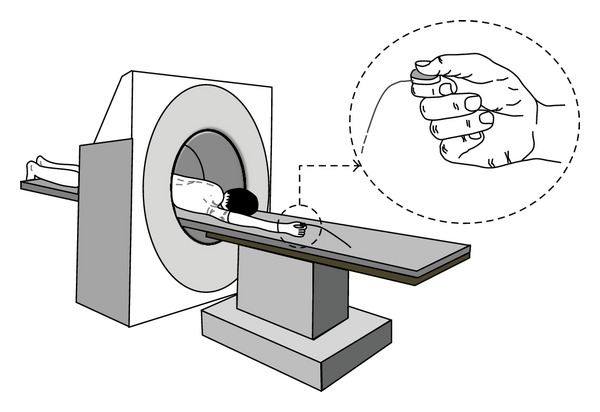
Testing position for the subjects in the CT machine and a custom jig containing a single-axis load cell, which were used to obtain the image and force data of the thumb, respectively.

A computed tomography (CT) scanner (Siemens Medical, Forchheim, Germany) and CARE Dose 4D software (Siemens Medical, Forchheim, Germany) were used to obtain 3D images of the focal thumb region, which ranged from the tip of the thumb to the trapezium. A single-axis load cell (SLB-25, Transducer Techniques, Inc., CA, USA) embedded in a self-designed jig was used to standardize the measurement of the lateral pinch performance and quantify the pinch forces (Figure 1). Signals from the load cell were amplified and sampled at 100 Hz using a 12-bit resolution A/D converter via the InstruNet data acquisition box (Model 230, National Instruments, Texas, USA), and the data were collected and displayed using a tablet computer. After obtaining the CT images, the Avizo 6 software (BSG SAS, Bordeaux, France) was used to reconstruct the 3D model of the thumb from the sliced images.

### 2.3. Acquisition Procedure

The subject's posture for the tests was lying prone on the bed of the CT machine, with the testing hand raised over the head with proper support to eliminate unnecessary movements during image taking (Figure 1). Before taking CT images, the maximum key pinch force of each subject was individually measured by the thumb contacted with the following three locations: the midpoint of the distal phalange (DP), proximal interphalangeal (PIP) joint, and the metacarpophalangeal (MP) joint of the index finger. Afterwards, a CT image of the hand was taken in a resting position, so that there was a slight wrist extension and finger flexion with the hand in a state of total relaxation. The subject then used the lateral pinch performance of their thumb and index finger to hold the jig with the load cell in one of three pinch locations, which were randomly assigned. For each pinch location, each subject took pinch force tests following the sequence of 0%, 50%, and 80% of individual maximum pinch force. The subject was asked to exert pinch force continuously between two force tasks, and no rest period was allowed to avoid changes in pinch posture.

### 2.4. Data Analysis

The arthrokinematics of the TMC joint are determined based on how and where the 1st metacarpal bone moves relative to the trapezium. Cheze's method was modified in this study to construct the coordinate systems (CS) of the 1st metacarpal bone and the trapezium [21]. The metacarpal lateral distal tubercle (MLDT), the metacarpal medial distal tubercle (MMDT), and the geometric center of the metacarpal base (GMB) were chosen to establish the coordinate system of the 1st metacarpal bone. The geometric center of the trapezium and 1st metacarpal joint (G_TM1J), the trapezium distal ulnar end of tubercle (TDUET), the trapezium distal radial end of tubercle (TDRET), and the dorsal end were used to establish the coordinate system of the trapezium. All the tasks associated with bony landmark digitization were carried out three times by the same person in order to avoid errors.

After setting up the coordinate system of the bony segments, the displacement of the articular surface center of the 1st metacarpal bone, selected as the reference point, was calculated as joint gliding relative to the trapezium. The gliding distance in three directions was normalized using the length, width, and height of the trapezium and defined as the gliding ratio. The coordinates of the nodes of the 3D model were employed to calculate the kinematic parameters of the thumb using MATLAB R2007b (The Mathworks, Natick, MA, USA).

### 2.5. Statistical Analysis

Statistical analysis was performed using the SPSS 17.0 statistical software (SPSS Inc., Chicago, Illinois, USA). Two-way ANOVA with repeated measures was employed to determine the effects of different pinch locations as well as the pinch forces on the characteristics of joint gliding. One-way ANOVA with repeated measures was used to examine the single main effect. The level of significance was set at *P* < 0.05. Post hoc analysis was then executed using the Bonferroni method to ensure interval adjustment. All the data were expressed as means and standard deviations.

## 3. Results

**Figure 2:**
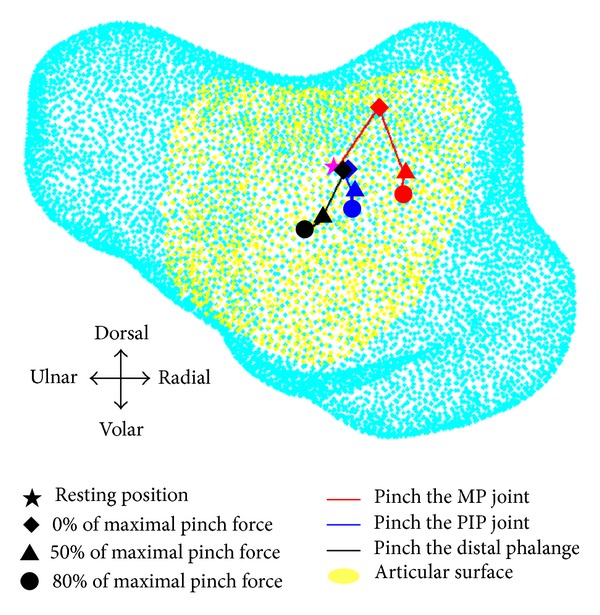
The gliding path of three different pinching conditions, MP joint (red), PIP joint (blue), and DP (black), with different levels of applied force.

Figure 2 shows representative locations of the reference points when performing the lateral pinch at different locations with different forces. When the MP joint is pinched, the reference points seem to move towards the dorsal-radial direction from the resting position and glide towards the volar direction when there is an increase in force. Then, when the PIP joint is pinched, the reference points move in a similar pattern as when the MP joint is pinched, but the gliding motion towards the radial side is less than that of the MP joint. Last, pinching the DP causes the reference points to glide toward the volar-ulnar direction from the resting position. However, the joint glides towards the dorsal-radial direction when the pinch force increases.

**Figure 3:**
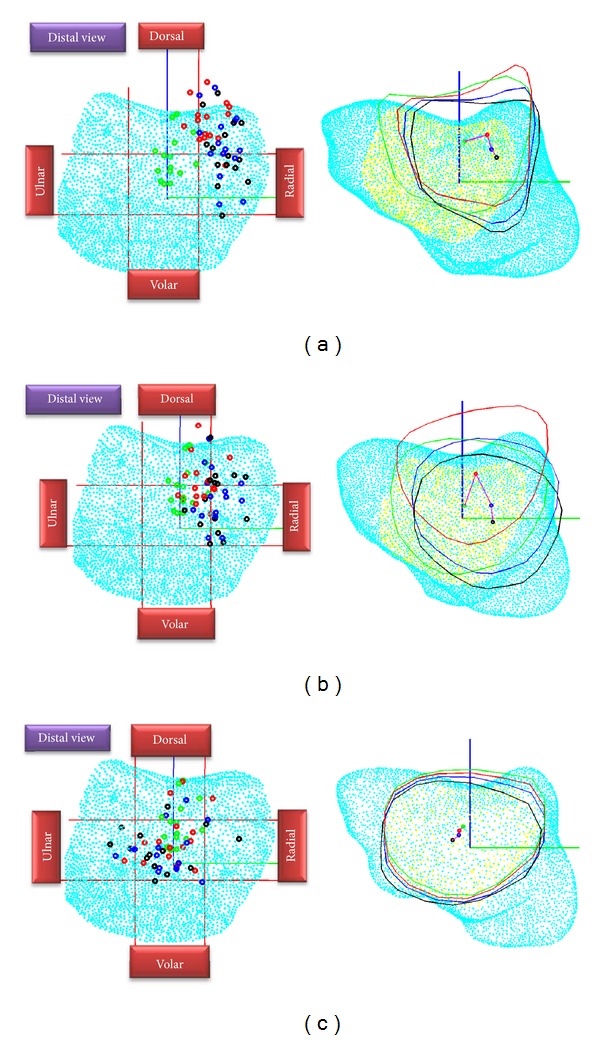
The distribution of the reference points at different pinching locations with three forces. The resting reference points are green, the points without force are red, the points with 50% maximum force are blue, and the points with 80% maximum force are black. The distribution of all subjects (left) and average trajectory of reference point movement (right) are shown above. The figures for when the MP joint, PIP joint, and DP are pinched are labeled (a), (b), and (c), respectively.

The distribution of reference points from all subjects and the trajectory of points when pinching the MP joint, PIP joint, and DP are shown in Figure 3. At the resting posture, the reference points are mainly located in the central and dorsal region. When the MP joint is pinched, the reference points move towards the dorsal-radial direction. However, when the pinch force increases, the reference points move towards the volar direction and are located in the dorsal-radial region. The distributions of the reference points when pinching the PIP joint are similar to those during pinching the MP joint but are less consistent among subjects. However, compared to pinching the MP joint, the reference points do not move as much towards the radial direction. These reference points are located in the center and dorsal region when no pinch force is applied. However, when force is applied, most points move towards the center and radial regions. When pinching the DP, the reference point distribution, compared to that seen with other pinching locations, is inconsistent among subjects. Nevertheless, most of the reference points move from the center region towards the volar-ulnar direction as the pinching force increases.

**Figure 4:**
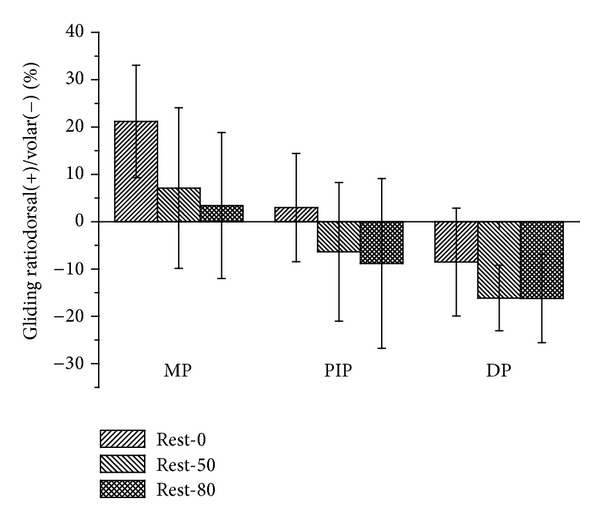
The gliding ratio of the reference points in the dorsal-volar direction represented in the coordinate system of the trapezium.

Three components of gliding ratios relative to the CS of the trapezium in the resting position at different pinching locations, as well as different pinching forces, are shown in Table 1. The effects of different pinching locations and pinching forces on the gilding ratios are indicated in the ANOVA table (Table 2). The main effect of both pinching location and force magnitude on the gliding ratio in the radial-ulnar direction can be seen in the results and only that of the force magnitude on the gliding ratio in the distal-proximal direction (*P* < 0.001). The interaction effect of pinching location and force magnitude on the gliding ratio is shown in both the dorsal-volar and distal-proximal directions (*P* < 0.001). In the dorsal-volar direction (Figure 4), the gliding ratios while pinching the MP joint decrease when the pinching force increases. The ratio reaches statistical significance when the MP joint is pinched with forces that range from resting to 80% (*F* = 3.78, *P* = 0.03). The gliding ratio increases along with the pinching force when pinching the PIP joint. However, the changes of gliding ratio from dorsal to volar direction were without statistical significances when the pinch force was applied. With regard to pinching the DP, the gliding ratio also increases along with the pinching force in the volar direction, although this result is also not statistically significant. When pinching 0% of individual maximum pinch force, the gliding ratio at the MP position is significantly larger than at the PIP joint and the DP (*F* = 9.24, *P* < 0.001).

**Figure 5:**
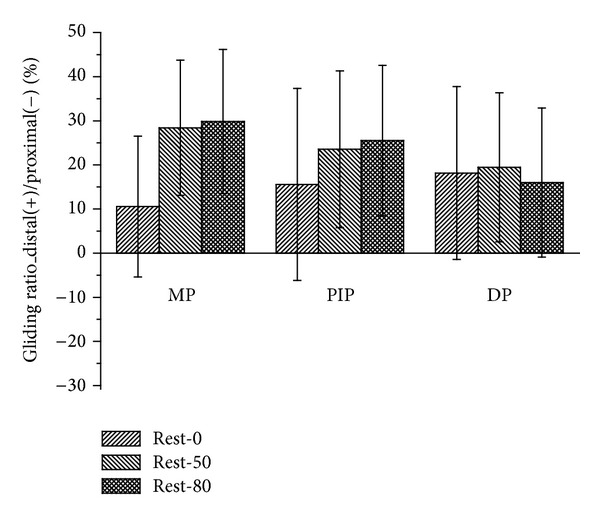
The gliding ratio of the reference points in the distal-proximal direction represented in the coordinate system of the trapezium.

In the distal-proximal direction, gliding ratio increases along with the increased pinching force when pinching the MP and PIP joints (Figure 5). Only when the MP joint is pinched does the gliding ratio reach a statistically significant level of rest-50% and rest-80% force (*P* < 0.05). No significant difference is seen when pinching the PIP joint and DP.

**Figure 6:**
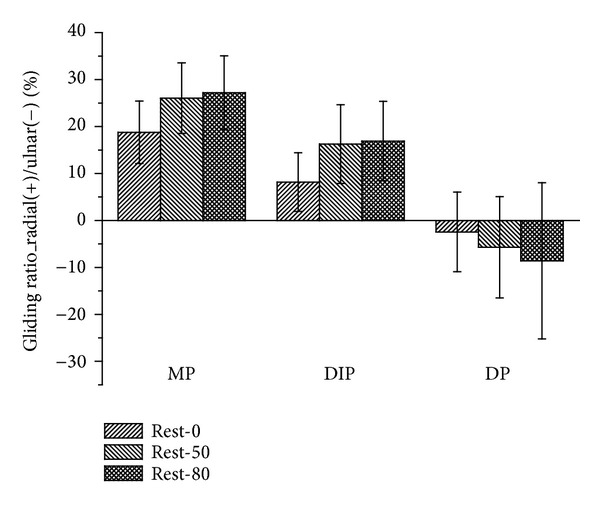
The gliding ratio of the reference points represented in the coordinate system of the trapezium.

In the radial-ulnar direction, the gliding ratio increases along with the increased pinching force (Figure 6). This occurs when pinching the MP and PIP joints, as well as the DP. When pinching the MP and PIP joints, the gliding ratios of rest-50% and rest-80% forces reach statistical significance (*P* < 0.05), while a significant difference is observed in the rest-50% pinching force when pinching the DP. The reference points glide toward the radial direction when pinching the MP and PIP joints but glide toward the ulnar direction when pinching the DP.

## 4. Discussion

In previous studies, efforts were made to find the contact patterns of joint gliding by simulating functional pinching in cadaver models [8, 18, 22]. Joint gliding was also measured using 2D models and with stress radiography in passive motions [17, 23, 24]. However, it is important to maintain joint stability during active motions, and these earlier works were able to explain little with regard to this.

In the current study, joint gliding during active functional pinching was measured with a 3D model. Three pinching locations were considered, for which there are reference points that move from the central region to the dorsal-radial region when changing from pinching the distal phalange to the MP joint without pinching force being applied. The reference points also slide towards the volar-ulnar direction when the pinching force increases. Overall, when pinching the MP joint without pinching force being applied, the reference points are located at the most dorsal-radial region. In contrast, the reference points when pinching the distal phalange with 80% of the maximum pinching force are the closest to the volar-ulnar direction (Figure 2). In the dorsal-volar direction, the reference points are located on the dorsal side in pinching locations with no pinching force, but these points will slide to the volar direction when a pinching force is applied (Figure 2).

The gliding ratio does not have any statistically significant difference when between 50% and 80% of the maximum force is applied in the dorsal-volar direction. Therefore, we suggest that the joint gliding that occurs near the end range in a pinching motion has about 50% of the force that is applied to the normal TMC joint. This means that an increase in pinching force will not make the joint glide even more. This phenomenon was not found when pinching the DP, but instead the gliding ratio only reached statistical significance when between 0% and 50% of the force was applied. We think that, when pinching the DP of the index finger, the metacarpophalangeal and interphalangeal joints of the thumb become more moveable, thus influencing the consistency of the results. Moulton's study in 2001 found that different ranges of motion of the metacarpophalangeal joint of the thumb would affect the TMC joint's contact patterns [25]. In other words, the range of motion of the metacarpophalangeal joint of the thumb may influence the joint gliding distance and ratio.

**Figure 7:**
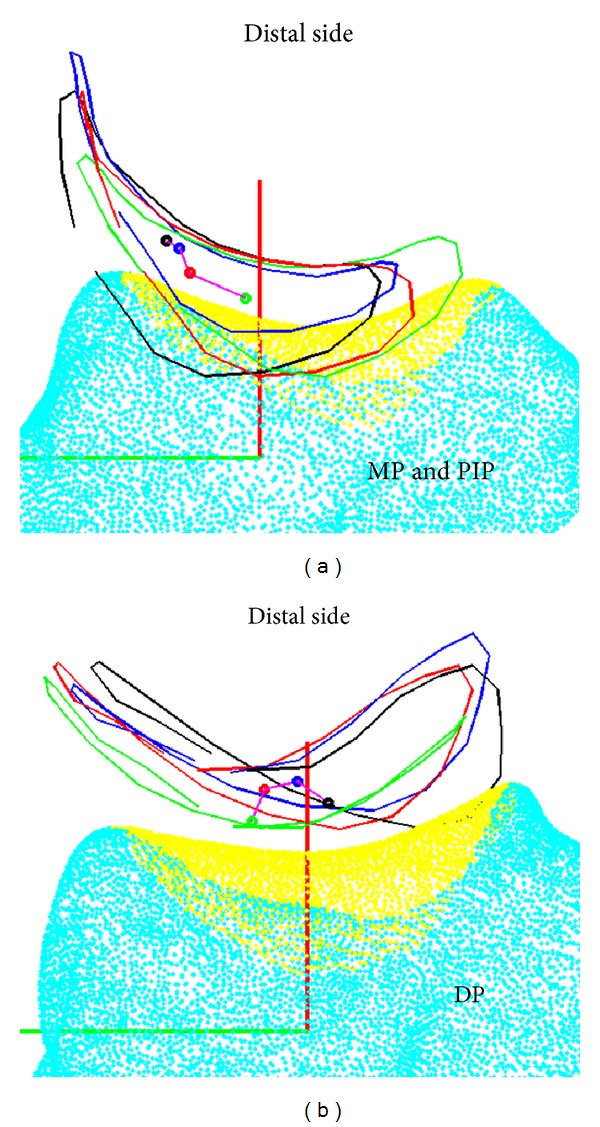
The gliding path of the lateral pinch from the dorsal view: (a) path for pinching the MP and PIP joints and (b) path for pinching the DP.

In the distal-proximal direction, the reference points slide to the distal direction when there is an increase in the pinching force that is applied on the MP and PIP joints. When the DP is pinched, the reference points glided towards the distal side without any force. When 50% force is applied on the joint, the gliding ratio is actually larger in the dorsal direction, as opposed to when 80% force is applied, which leads to a smaller gliding ratio. Clinically, the two bones of the finger joint should be closer when the compression force increases. However, our results do not support this. Our results indicate that the gliding path is relative to the CS of the trapezium when pinching the MP joint, PIP joint, and DP with three pinching forces (Figure 7). The shape of the articular surface of the trapezium (yellow region) is concave in the radial-ulnar direction and convex in the dorsal-volar direction, which is consistent with the results in Humes et al. [26]. When pinching the MP and PIP joints, the reference points glide towards the radial side with more pinching force. Furthermore, the radial side of the articular border is higher than the articular center, because of the concave shape of the articular surface. It is thus inferred that the reference points may slide towards the distal direction when the MP and PIP joints are pinched, because of the shape of the articular surface. When the DP is pinched, the reference points may glide towards the volar-ulnar direction (from the green point to the black point in Figure 7(b)). This thus shows that the gliding path is influenced by the articular shape of the bone. In addition, 50% of the force is applied when the reference points are the nearest to the distal direction.

The gliding ratio when the MP joint is pinched reaches a value of statistical significance between rest-50% and rest-80% in the distal-proximal direction. When the PIP joint is pinched, the gliding ratio grows towards the distal direction as force increases. When pinching both the MP and PIP joints, the reference points slide towards the distal end because of an increase in pinch force. However, the gliding ratios still do not reach statistical significance. The reference points may slide towards the maximum range at 50% force, and thus the gliding ratio may not increase with 80% force. In addition, the gliding path of the reference points when pinching the DP is not the same as those when pinching the MP and PIP joints and does not reach any statistical significance, because pinching the DP is influenced by the articular surface of the trapezium.

In the radial-ulnar direction, the reference points slide towards the radial direction as the pinch force increases on the MP and PIP joints. This motion produces gliding ratios that reach statistical significance between rest-50% and rest-80% force. When pinching the DP, the reference points slide towards the ulnar direction, which is opposite to what is seen when pinching the MP and PIP joints, causing the gliding ratio to increase along with the pinching force. Therefore, we infer that the metacarpophalangeal and interphalangeal joints of the thumb have smaller constraints when the DP is pinched, and thus the metacarpophalangeal joint of the thumb will extend, letting the reference points move toward the volar-ulnar side [24]. Furthermore, the results also show that when 50% force is applied to the TMC joint it is in stable condition, and thus an increase in pinch force does not influence how far the reference points glide.

The following limitations of this study should be noted. We used CT images to measure the joint displacement during pinching with different applied forces, and thus the radiation exposure is the fact for the subject when performing the tasks. However, we have tried the best to reduce the possibility of radiation injury as much as possible for the subjects through detailed and careful estimation of the radiation. In addition, the posture of the thumb during pinching may vary between subjects because of different habits with regard to performing this action, and this might lead to significant variations with regard to joint gliding.

In summary, the results of this study highlight some further potential applications. From biomechanical viewpoint, an understanding of TMC joint kinematics *in vivo* may provide more valuable and realistic information with regard to the joint than what an *in vitro* study did. While this study has indicated the effect of thumb posture and applied force on the TMC joint kinematics, future biomechanical research still should consider the effect of other anthropometric and ergonomic factors on the movement characteristics of this joint. A thorough comprehension of the joint characteristics when performing pinching tasks may have practical benefits for clinicians to educate their patients on utilizing suitable joint protection strategies while doing manipulative tasks to prevent the joint from OA interference. In addition, the knowledge of joint kinematics may also provide worthy information with regard to designing and manufacturing suitable TMC joint implants as well as hand tools further.

## Figures and Tables

**Table 1:** Descriptive data of gliding ratio relative to the CS of the trapezium in the resting position at different pinching locations as well as different pinching forces.

Variables	MP			PIP			DP		
	Rest-0	Rest-50	Rest-80	Rest-0	Rest-50	Rest-80	Rest-0	Rest-50	Rest-80
Dorsal/volar	0.22 (0.10)	0.15 (0.09)	0.13 (0.09)	0.09 (0.07)	0.12 (0.10)	0.15 (0.12)	0.11 (0.09)	0.16 (0.07)	0.17 (0.08)
Distal/proximal	0.16 (0.11)	0.28 (0.15)	0.30 (0.16)	0.22 (0.14)	0.27 (0.12)	0.26 (0.16)	0.20 (0.18)	0.21 (0.15)	0.18 (0.14)
Radial/ulnar	0.19 (0.07)	0.26 (0.07)	0.27 (0.08)	0.08 (0.06)	0.16 (0.08)	0.16 (0.08)	0.07 (0.06)	0.10 (0.07)	0.15 (0.11)

Data representation: mean (SD).

**Table 2:** Two-way repeated measure ANOVA table to determine the effect of different pinch locations as well as the pinch forces on the characteristics of joint gliding.

Variables	Joint		Force		Joint × Force		Post hoc of joint	Post hoc of force
	*F *	*P *	*F *	*P *	*F *	*P *		
Dorsal/volar	1.70	0.20	0.21	0.81	7.57	<0.00***	—	—
Distal/proximal	3.66	0.45	7.09	<0.00**	4.73	<0.00**	—	—
Radial/ulnar	18.19	<0.00***	38.57	<0.00***	2.25	0.08	MP > PIP, MP > DP	rest-0 < rest-50

*P < 0.05, **P < 0.01, ***P < 0.001.

## References

[1] Marzke MW (1997). Precision grips, hand morphology, and tools. *American Journal of Physical Anthropology*.

[2] Moran SL, Berger RA (2003). Biomechanics and hand trauma: what you need. *Hand Clinics*.

[3] Kuo L-C, Chiu H-Y, Chang C-W, Hsu H-Y, Sun Y-N (2009). Functional workspace for precision manipulation between thumb and fingers in normal hands. *Journal of Electromyography and Kinesiology*.

[4] Li Z-M, Tang J (2007). Coordination of thumb joints during opposition. *Journal of Biomechanics*.

[5] Lin H-T, Kuo L-C, Liu H-Y, Wu W-L, Su F-C (2011). The three-dimensional analysis of three thumb joints coordination in activities of daily living. *Clinical Biomechanics*.

[6] Youngleson JH (1965). The management of the contracted first web space. *South African Medical Journal*.

[7] Cooney WP, Chao EYS (1977). Biomechanical analysis of static forces in the thumb during hand function. *Journal of Bone and Joint Surgery A*.

[8] Ateshian GA, Ark JW, Rosenwasser MP, Pawluk RJ, Soslowsky LJ, Mow VC (1995). Contact areas in the thumb carpometacarpal joint. *Journal of Orthopaedic Research*.

[9] Cerveri P, de Momi E, Marchente M (2008). In vivo validation of a realistic kinematic model for the trapezio-metacarpal joint using an optoelectronic system. *Annals of Biomedical Engineering*.

[10] Cooney WP, Lucca MJ, Chao EYS, Linscheid RL (1981). The kinesiology of the thumb trapeziometacarpal joint. *Journal of Bone and Joint Surgery A*.

[11] Goubier J-N, Devun L, Mitton D, Lavaste F, Papadogeorgou E (2009). Normal range-of-motion of trapeziometacarpal joint. *Chirurgie de la Main*.

[12] Imaeda T, Cooney WP, Niebur GL, Linscheid RL, An K-N (1996). Kinematics of the trapeziometacarpal joint: a biomechanical analysis comparing tendon interposition arthroplasty and total-joint arthroplasty. *Journal of Hand Surgery*.

[13] Imaeda T, Niebur G, An K-N, Cooney WP (1994). Kinematics of the trapeziometacarpal joint after sectioning of ligaments. *Journal of Orthopaedic Research*.

[14] Imaeda T, Niebur G, Cooney WP, Linscheid RL, An K-N (1994). Kinematics of the normal trapeziometacarpal joint. *Journal of Orthopaedic Research*.

[15] Koff MF, Zhao KD, Mierisch CM, Chen M-Y, An K-N, Cooney WP (2007). Joint kinematics after thumb carpometacarpal joint reconstruction: an in vitro comparison of various constructs. *Journal of Hand Surgery*.

[16] Uchiyama S, Cooney WP, Niebur G, An K-N, Linscheid RL (1999). Biomechanical analysis of the trapeziometacarpal joint after surface replacement arthroplasty. *Journal of Hand Surgery*.

[17] Wolf JM, Oren TW, Ferguson B, Williams A, Petersen B (2009). The carpometacarpal stress view radiograph in the evaluation of trapeziometacarpal joint laxity. *Journal of Hand Surgery*.

[18] Koff MF, Shrivastava N, Gardner TR, Rosenwasser MP, Mow VC, Strauch RJ (2006). An in vitro analysis of ligament reconstruction or extension osteotomy on trapeziometacarpal joint stability and contact area. *Journal of Hand Surgery*.

[19] Brunelli G, Monini L, Brunelli F (1989). *Stabilisation of the Trapezio-Metacarpal Joint*.

[20] Xu L, Strauch RJ, Ateshian GA, Pawluk RJ, Mow VC, Rosenwasser MP (1998). Topography of the osteoarthritic thumb carpometacarpal joint and its variations with regard to gender, age, site, and osteoarthritic stage. *Journal of Hand Surgery*.

[21] Cheze L, Dumas R, Comtet JJ, Rumelhart C, Fayet M (2009). A joint coordinate system proposal for the study of the trapeziometacarpal joint kinematics. *Computer Methods in Biomechanics and Biomedical Engineering*.

[22] Pellegrini VD, Olcott CW, Hollenberg G (1993). Contact patterns in the trapeziometacarpal joint: the role of the palmar beak ligament. *Journal of Hand Surgery*.

[23] Eaton RG, Littler JW (1973). Ligament reconstruction for the painful thumb carpometacarpal joint. *Journal of Bone and Joint Surgery A*.

[24] Grood ES, Suntay WJ (1983). A joint coordinate system for the clinical description of three-dimensional motions: application to the knee. *Journal of Biomechanical Engineering*.

[25] Moulton MJR, Parentis MA, Kelly MJ, Jacobs C, Naidu SH, Pellegrini VD (2001). Influence of metacarpophalangeal joint position on basal joint-loading in the thumb. *Journal of Bone and Joint Surgery A*.

[26] Humes D, Jähnich H, Rehm A, Compson JP (2004). The osteology of the trapezium. *Journal of Hand Surgery*.

